# Angiotensin II Type 1 Receptor Blockers Inhibit KAT II Activity in the Brain—Its Possible Clinical Applications

**DOI:** 10.1007/s12640-017-9781-2

**Published:** 2017-07-21

**Authors:** Izabela Zakrocka, Katarzyna M. Targowska-Duda, Artur Wnorowski, Tomasz Kocki, Krzysztof Jóźwiak, Waldemar A. Turski

**Affiliations:** 1 Department of Experimental and Clinical Pharmacology, Medical University of Lublin, Jaczewskiego 8b, 20-090 Lublin, Poland; 2 Department of Biopharmacy, Medical University of Lublin, Chodźki 4a, 20-093 Lublin, Poland

**Keywords:** Kynurenic acid, Renin-angiotensin system, Angiotensin II type 1 receptor blockers, Arterial hypertension, Dementia, Schizophrenia

## Abstract

Angiotensin II receptor blockers (ARBs) are one of the most frequently recommended antihypertensive drugs. Apart from their activity towards the circulatory system, ARBs also penetrate the blood-brain barrier and display neuroprotective effects. Kynurenic acid (KYNA) is an endogenous metabolite of tryptophan produced by kynurenine aminotransferase II (KAT II) in the brain. Antagonism towards all ionotropic glutamate (GLU) receptors is the main mechanism of KYNA action. An elevated brain level of KYNA is linked with memory impairment and psychotic symptoms. The aim of this study was to examine the influence of three ARBs: irbesartan, losartan, and telmisartan on KYNA production and KAT II activity in rat brain. The effect of ARBs on KYNA production was analyzed in rat brain cortical slices and on isolated KAT II enzyme. Irbesartan, losartan, and telmisartan decreased KYNA production and KAT II activity in a dose-dependent manner in rat brain cortex in vitro. Molecular docking suggested that the examined ARBs could bind to an active site of KAT II. In conclusion, ARBs decrease KYNA production in rat brain by direct inhibition of KAT II enzymatic activity. This novel mechanism of ARBs action may be advantageous in the treatment of cognitive impairment or the management of schizophrenia.

**Electronic supplementary material:**

The online version of this article (doi:10.1007/s12640-017-9781-2) contains supplementary material, which is available to authorized users.

## Introduction

Arterial hypertension remains the most common cardiovascular disorder affecting nearly half of the population (ESH/ESC Task Force for the Management of Arterial Hypertension 2013). The prevalence of hypertension is closely related with the occurrence of stroke, myocardial infarction, kidney failure, and higher mortality risk (Mankin 2016). Despite a variety of antihypertensive drugs being available, an appropriate blood pressure control is still difficult to achieve in a large group of patients with arterial hypertension (Sarganas and Neuhauser 2016). According to the guidelines, renin-angiotensin system (RAS) inhibitors are the most preferred hypotensive agents. With the exception of a decrease in blood pressure, their antiinflammatory and antioxidative properties are responsible for end-organ protection and mortality reduction (Muñoz-Durango et al. 2016).

Studies on RAS revealed the occurrence of tissue RAS and its paracrine function (Baltatu et al. 2011). The presence of RAS in the brain began to attract the attention of neuroscientists. First information about renin-like enzyme forming angiotensin in the brain was published by Ganten et al. (1971). Apart from its role in water and electrolyte homeostasis, brain RAS is linked with the development of epilepsy (Pereira et al. 2010), Alzheimer’s disease (AD) (Hajjar and Rodgers 2013), Parkinson’s disease (Labandeira-García et al. 2014), and neuropathic pain (Muthuraman and Kaur 2016). Active components of RAS are synthesized from angiotensinogen present primarily in glial cells (Intebi et al. 1990). The main receptors responsible for angiotensin II (AT-II) action are AT-II type 1 receptors (AT_1_R) which dominate in astroglial cells (Sumners et al. 1994). Activation of central AT_1_R by AT-II is linked with the pathogenesis of hypertension (Toney and Porter 1993). Reduction of AT-II synthesis and inhibition of AT_1_R are the main goals of antihypertensive therapy. Since other enzymes, e.g., tonin, may produce AT-II from angiotensin I or angiotensinogen (Kondo et al. 1980), AT_1_R blockers (ARBs) seem to provide better control over RAS activity than angiotensin converting enzyme (ACE) inhibitors.

Kynurenic acid (KYNA), an endogenous metabolite of tryptophan, was discovered in the nineteenth century in the dogs’ urine by Justus von Liebig (1853). In the brain, KYNA production from precursor kynurenine (KYN) takes place mainly in astrocytes (Guillemin et al. 2000). Among kynurenine aminotransferases (KAT) catalyzing KYNA synthesis, KAT II has a dominant role in this process (Nematollahi et al. 2016). It is well established that the main mechanism of KYNA action is the blockade of ionotropic ﻿﻿glutamate (GLU﻿) receptors﻿, *N*-methyl-d-aspartate (NMDA), α-amino-3-hydroxy-5-methyl-4-isoxazolepropionic acid (AMPA), and kainate (Schwarcz and Stone 2017). Non-competitive antagonism towards the α7 nicotinic acetylcholine receptors (Beggiato et al. 2013) or activation of G protein-coupled receptors 35 (GPR35) (Stone et al. 2013) are other effects of KYNA. GLU injected into the rostral ventrolateral medulla (RVLM) was shown to elevate blood pressure and heart rate in anesthetized rats (Willette et al. 1987). KYNA, as a GLU antagonist, was proven to lower blood pressure after central administration (Araujo et al. 1999; Ito et al. 2000).

Considering that ARBs have been shown to abolish central pressor GLU effect (Vieira et al. 2010), the goal of the present study was to examine the influence of three ARBs, irbesartan, losartan, and telmisartan, on KYNA synthesis and KAT II activity in rat brain cortex in vitro. In addition, the available crystal structure of the human KAT II (hKAT II) in complex with its substrate KYN and 4′-deoxy-4′-aminopyridoxal-5′-phosphate (PMP) enabled us to predict a possible binding site for the studied ARBs.

## Materials and Methods

### Animals

Experiments were performed on male Wistar rats (weight 150–200 g) obtained from an accredited breeder (Brwinów, Poland). Animals were kept under standard laboratory conditions at room temperature, 12-h light-dark cycles, and in cages with food and water available ad libitum. Procedures were conducted between 7 a.m. and 1 p.m. All animals were adapted to laboratory conditions for 7 days before tests were carried out. Procedures were accepted by the I Local Ethics Committee for Animal Experiments in Lublin and are in agreement with Directive 2010/63/EU on the protection of animals used for scientific purposes.

### Chemical Substances

The following chemicals were purchased from Sigma-Aldrich: l-kynurenine (sulfate salt), irbesartan, losartan potassium, telmisartan, dimethyl sulfoxide (DMSO), sodium chloride, potassium chloride, magnesium sulfate, calcium chloride, sodium phosphate monobasic, sodium phosphate dibasic, glucose, distilled water, Trizma base, acetic acid, pyridoxal 5′-phosphate, 2-mercaptoethanol, pyruvate, and glutamine. Substances of the highest purity used for high-performance liquid chromatography (HPLC) were obtained from J. T. Baker Chemicals and Sigma-Aldrich.

### Experiments Conducted on Cortical Slices

Experiments on cortical slices were performed as previously described by Turski et al. (1989). After the rats’ decapitation, their brains were removed from the skulls and placed on ice. Brain cortex was immediately separated from the white matter and cut with a McIlwain tissue chopper (Mickle Laboratory Engineering Co. Ltd., USA). Cortical slices (size 1 mm × 1 mm) were placed into incubation wells (10 slices in each well), filled with 1 ml of oxygenated Krebs-Ringer buffer at pH 7.4. The incubation lasted 2 h at 37°C in the presence of 10 μM l-KYN and one of four drug concentrations (0.01, 0.05, 0.1, or 1 mM). Minimum six wells were used to examine each drug concentration. The incubation was ended by placing the samples into an ice-cold bath. Obtained supernatants were centrifuged (15,133×*g* for 15 min) and applied to ion exchange resin Dowex 50 W+ column. Eluted KYNA was separated by the HPLC (Thermo Fisher Scientific HPLC system, ESA catecholamine HR-80, 3 μm, C18 reverse-phase column) and quantified fluorometrically. The resulting peak was compared with the authentic KYNA. Experiments were conducted at least three times to achieve comparable results.

### Evaluation of Kynurenine Aminotransferase Activity

Analysis of KAT II activity was performed according to the method by Guidetti et al. (1997). To examine KAT II activity, the brain cortex was homogenized in dialysate buffer made from 5 mM Tris-acetate buffer at pH 8.0, 50 μM pyridoxal 5′-phosphate, 10 mM 2-mercaptoethanol, and distilled water. Prepared homogenate was centrifuged (15,133×*g* for 15 min) and the supernatant dialyzed for 12 h at 8 °C using cellulose membrane dialysis tubing (dialysis tubing cellulose membrane, average flat width 10 mm; Sigma-Aldrich) in 4 l of the dialysate buffer. Afterwards, the enzyme preparation was incubated in the reaction mixture containing incubation solution, 2 μM l-KYN, and solutions of tested drugs (at 0.01, 0.05 0.1, and 1 mM concentration). The reaction pH was 7.0 (optimal pH for KAT II). l-glutamine was added to inhibit KAT I activity. Three probes were used for each drug concentration. The incubation lasted for 2 h at 37 °C and was ended by transferring the samples to an ice-cold bath. Supernatants were centrifuged and KYNA content analyzed, as described previously.

### Statistical Analysis

Data were presented as a percentage of control values. Mean and standard error of the mean (SEM) were calculated. Statistical analysis was performed using one-way analysis of variance (ANOVA) with a post hoc Tukey-Kramer test. *P* < 0.05 was considered statistically significant. All calculations were made with the GraphPad InStat program, version 3.06.

### Molecular Docking of ARBs and Kynurenine to KAT II

The available crystal structure of the hKAT II in complex with its substrate kynurenine and co-factor PMP at 1.95 Å atomic resolution (PDB ID: 2R2N) (Han et al. 2008) was used to perform the molecular docking. More specifically, each studied ligand (i.e., irbesartan, losartan, and telmisartan) (Molfile) was imported from the ChEMBL Database and optimized using the semi-empirical method AM1, and then transferred for the subsequent step of ligand docking. Molegro Virtual Docker (v 6.0.0, Molegro ApS, Aarhus, Denmark) was used for docking simulations of flexible ligands into the rigid KAT II target. The docking space (a sphere of 20 Å diameter) was defined to cover KYN (substrate), and the co-factor (PMP). KYN was then removed and each ARB was docked to the KAT II structure. The actual docking simulations were performed using the following settings: number of runs = 100, maximal number of poses returned = 10. Additional docking was performed for KYN to check the correctness of the docking procedure. The lower energy conformations were selected from each cluster of superposed poses for each studied ligand.

## Results

### Evaluation of KYNA Production in Brain Cortical Slices In Vitro

**Fig. 1:**
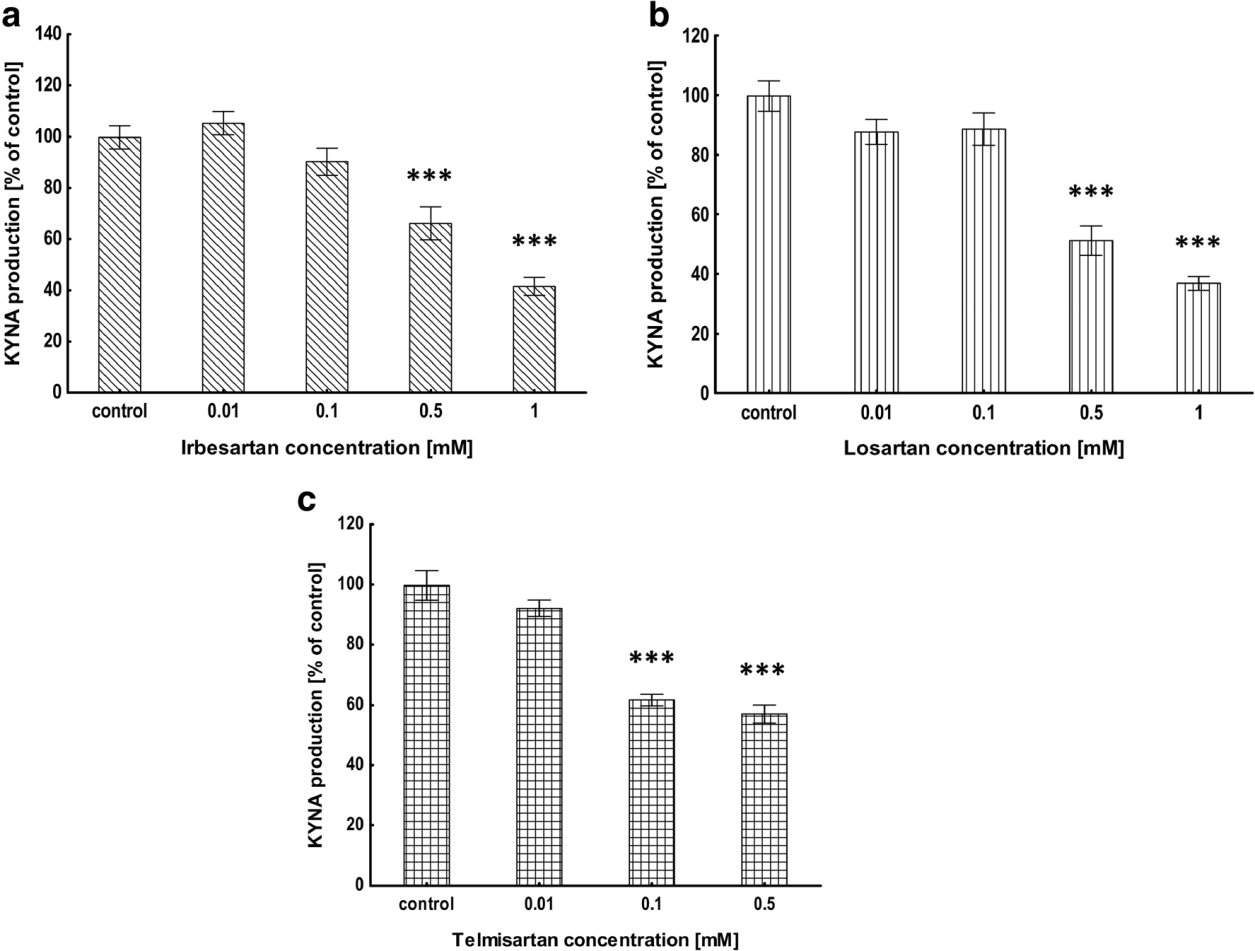
Influence of irbesartan (**a**), losartan (**b**) and telmisartan (**c**) on KYNA production in rat brain cortical slices in vitro. Data are expressed as a percentage of the control production, mean ± SEM, *n* = 6, ANOVA with post hoc Tukey-Kramer test, and *triple asterisks* indicate *P* < 0.001 vs. control

De novo production of KYNA in rat brain slices in vitro under standard conditions was 3.41 ± 0.07 pmol/well. All analyzed ARBs, irbesartan, losartan, and telmisartan decreased KYNA production in rat brain cortical slices in vitro (Fig. 1). At the concentration of 0.5 and 1 mM irbesartan decreased KYNA production to 66﻿﻿% (*P* < 0.001) and 42% (*P* < 0.001) of the control value, respectively (Fig. 1a). Losartan at the concentration of 0.5 and 1 mM inhibited KYNA synthesis to 51% (*P* < 0.001) and 37% (*P* < 0.001) of the control value, respectively (Fig. 1b). Telmisartan at 0.1 and 0.5 mM concentration decreased KYNA production to 62% (*P* < 0.001) and 57% (*P* < 0.001) of the control value, respectively (Fig. 1c).

### Evaluation of KAT II Activity in Brain Cortical Homogenates In Vitro

**Fig. 2:**
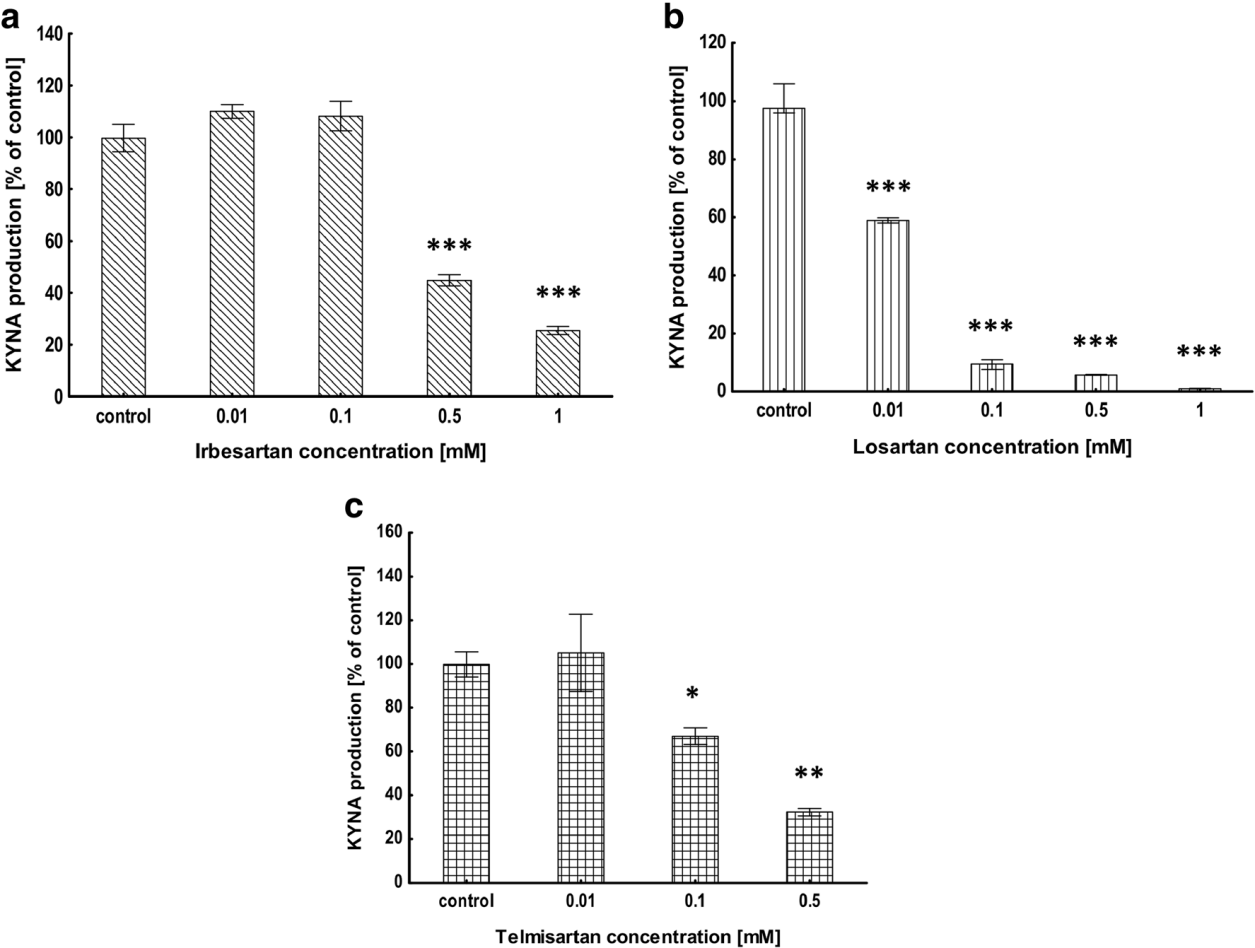
Influence of irbesartan (**a**), losartan (**b**) and telmisartan (**c**) on KAT II activity in rat brain cortex in vitro. Data are expressed as a percentage of the control KYNA production, mean ± SEM, *n* = 3, ANOVA with post hoc Tukey-Kramer test, and *single asterisk* indicates *P* < 0.05 vs. control, *double asterisks* indicate *P* < 0.01 vs. control, and *triple asterisks* indicate *P* < 0.001 vs. control

The mean activity of KAT II under standard conditions was 19.17 ± 1.15 pmol of KYNA per test tube. Irbesartan at 0.5 and 1 mM concentration inhibited KAT II in rat brain cortical homogenates in vitro to 4﻿5% (*P* < 0.001) and 25% (*P* < 0.001) of the control value, respectively (Fig. 2a). Losartan decreased KAT II activity in rat brain cortical homogenates in vitro at the concentration of 0.01, 0.1, 0.5, and 1 mM to 59% (*P* < 0.001), 10% (*P* < 0.001), 6% (*P* < 0.001), and 1% (*P* < 0.001) of the control value, respectively (Fig. 2b). Telmisartan at 0.1 and 0.5 mM concentration decreased KAT II activity in rat brain cortical homogenates in vitro to 63% (*P* < 0.05) and 32% (*P* < 0.01) of the control value, respectively (Fig. 2c).

### Molecular Docking of ARBs and Kynurenine to KAT II

**Fig. 3:**
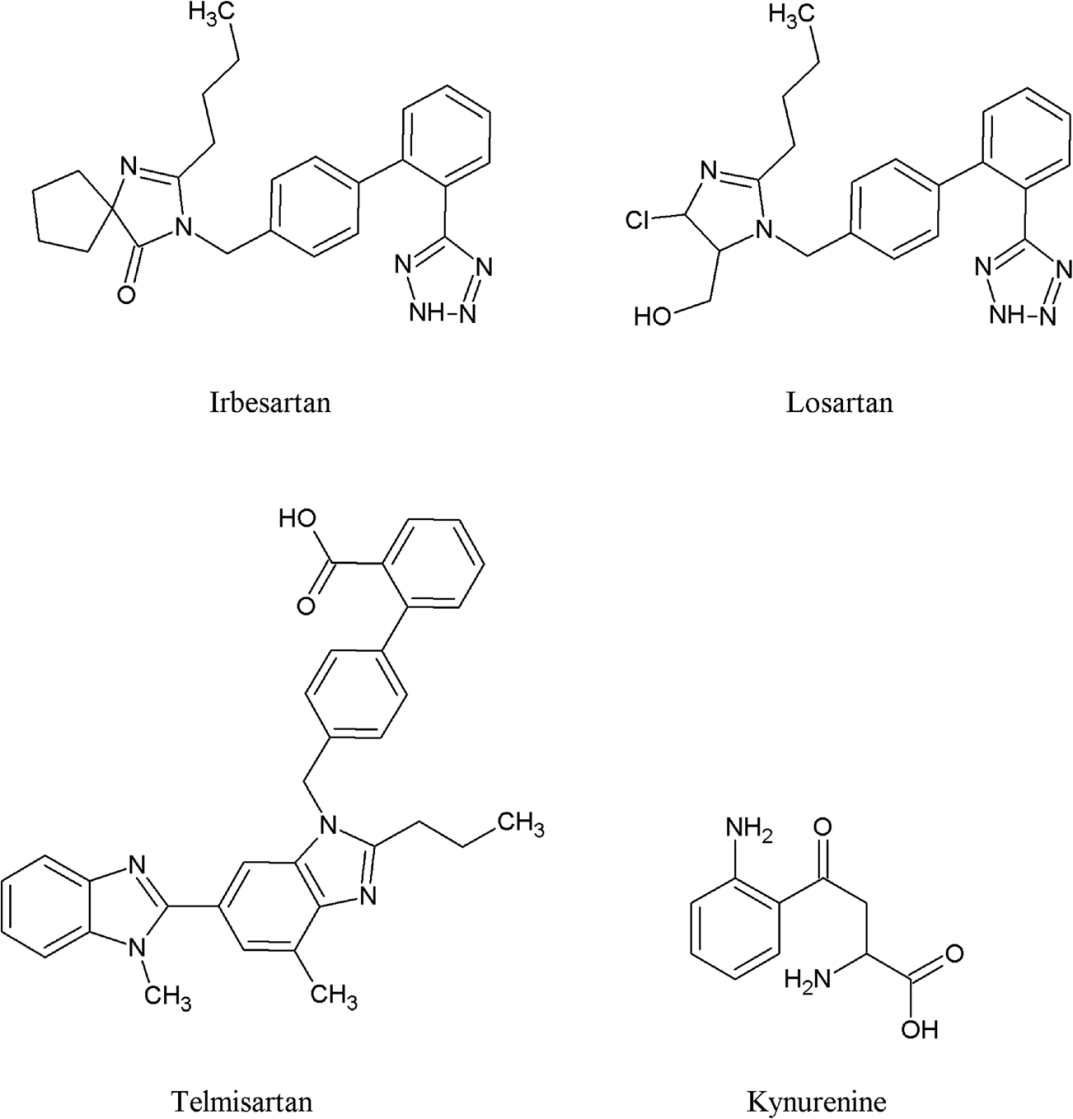
Molecular structures of ARBs (i.e., irbesartan, losartan, and telmisartan) and KYN (physiological KAT II substrate)

**Fig. 4:**
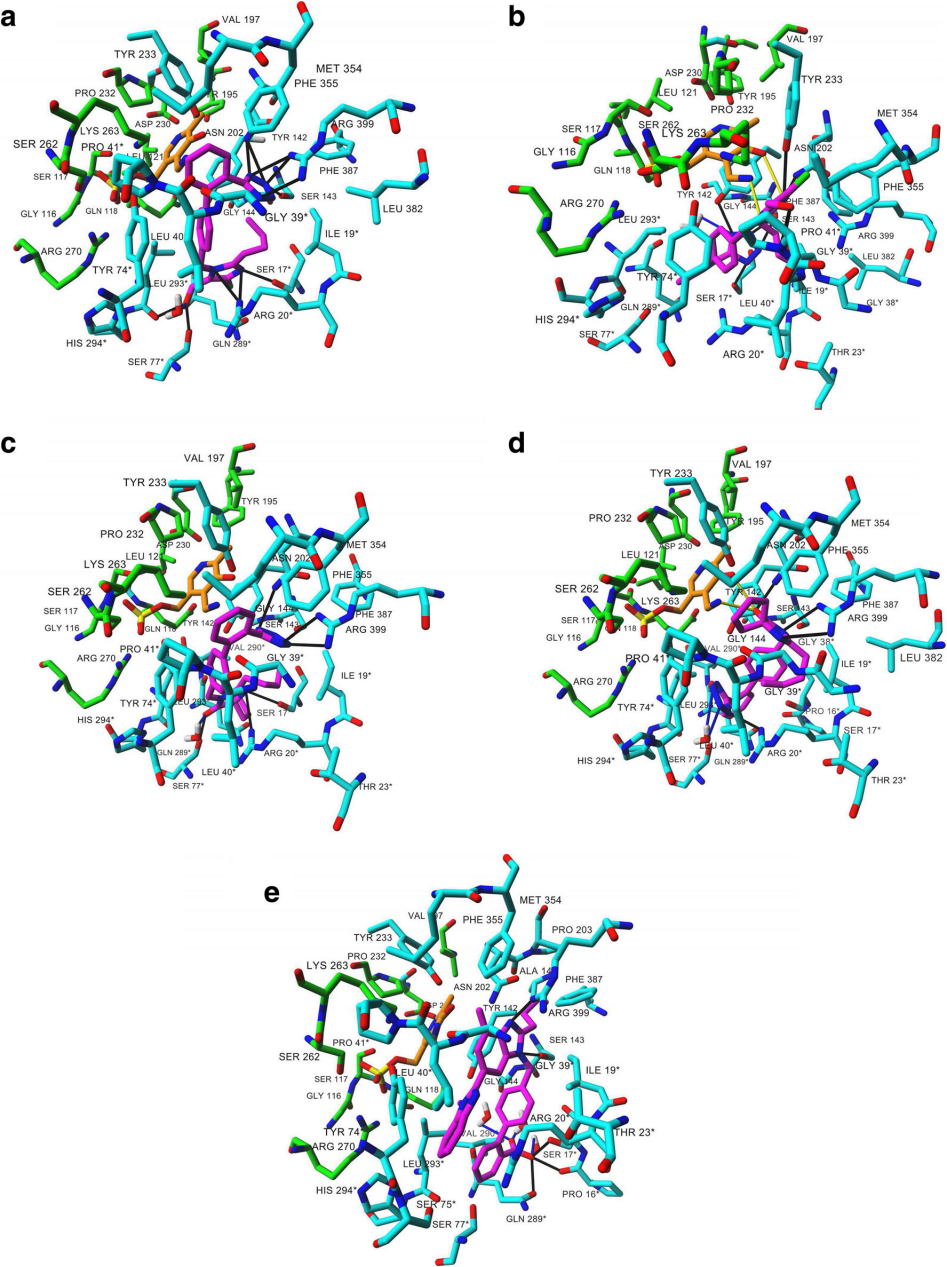
Molecular docking of losartan, irbesartan, and telmisartan to the crystal structure of KAT II (PDB ID: 2R2N). All ligand binding sites overlap the KYN binding pocket. Two different ligand orientations are suggested for losartan (**a**, **b**) and irbesartan (**c**, **d**), and one for telmisartan (**e**). Ligand and co-factor are rendered in stick mode; residues involved in each ligand and PMP binding are shown in *cyan* and *green*, respectively. Residues from chain A are labeled with an *asterisk* to differentiate chain A from chain B residues. *Black solid lines* indicate hydrogen bonds as well as salt bridges formed between each ligand and amino acid residues, *blue solid lines* between ligands and water molecules, and *yellow solid lines* indicate the hydrogen bonds formed between losartan (**b**) or irbesartan (**d**) and the co-factor. All residues involved in hydrogen bonding are marked in *red*. Oxygen atoms are colored *red*, nitrogens *blue*, phosphorus *yellow*, and chlorine *green*. All hydrogen atoms are hidden (color figure online)

The molecular docking results showed that each studied ARB (structures presented in Fig. 3) binds to the KAT II active site. In addition, our results suggested a similar KYN orientation within the KAT II active site as determined in the three-dimensional crystal structure (Han et al. 2008) of KAT II with KYN (PDB ID: 2R2N). This confirmed the correctness of the docking procedure. Our findings suggest two orientations of losartan and irbesartan at the enzyme active site, and one proposal for telmisartan (Fig. 4 and Table S1). More specifically, in both orientations, losartan interacts with the residues indicated for KYN, including Ile19 (A), Arg20 (A), Gly39 (A), Leu40 (A), Tyr74 (A), Leu293 (A) from one subunit, and Tyr142 (B), Ser143 (B), Asn202 (B), Tyr233 (B), Phe355 (B), Phe387 (B), and Arg399 (B) from the opposite subunit. In addition, this molecule interacts with additional residues (mostly the same for both orientations). In orientation 1, the hydrogen bond interactions suggested by the docking are formed between the tetrazole moiety of losartan and Asn202 (B) Ser143 (B), and Arg399 (B); between losartan imidazole moiety and Ser17 (A), Arg20 (A), Ser77 (A), and Leu293 (A); as well as between losartan and water molecule (Fig. 4a and Table S1). In orientation 2, tetrazole moiety is oriented in the opposite direction compared to orientation 1. More specifically, the hydrogen bonds are formed between tetrazole moiety of losartan and Ser17 (A), Thr142 (B), and Ser143, and between losartan imidazole moiety and Asn202 (B), Gly39 (A), Pro41 (A), and Tyr233 (B), as well as between losartan and water molecule (Fig. 4b). In orientation 2, two additional hydrogen bonds are suggested between losartan and PMP (co-factor) bound to the KAT II active site (Fig. 4b and Table S1).

Results of the molecular docking indicate almost the same orientations of irbesartan (Figs. 3 and 4c) within KAT II active site as described for losartan (Figs. 3 and 4a). In orientation 1, the same residues are shown for irbesartan as for losartan binding, whereas a reduced number of hydrogen bonds are suggested. In particular, the hydrogen bonds are formed between tetrazole moiety of irbesartan and Asn202 (B) and Arg399 (B); between irbesartan imidazole moiety and Ser17 (A), Ser77 (A), and Arg20 (A); as well as between irbesartan and water molecule (Fig. 4c and Table S1). Although two additional hydrogen bonds are suggested between irbesartan (in orientation 2) and PMP (co-factor) (Fig. 4d and Table S1), the reduced number of hydrogen bonds [i.e., one between tetrazole moiety of the ligand and Arg20 (A), and between ligand imidazole moiety and Arg399 (B) and Asn202 (B) (Fig. 4d)] is suggested for irbesartan bound to the KAT II active site.

Finally, the molecular docking data suggest that telmisartan binds to the same site as previously indicated for losartan and irbesartan at the KAT II active site. However, there are few more residues not involved in losartan and irbesartan binding. In addition, a lower number of hydrogen bonds is suggested, including that formed between the ligand and Pro16, Ser17, Ser143, and Arg399 (Fig. 4e and Table S1).

## Discussion

The present study shows that all examined ARBs, irbesartan, losartan, and telmisartan, reduce KYNA production in brain cortical slices in vitro. Moreover, all analyzed ARBs decrease the activity of KAT II in brain cortical homogenates in vitro. KAT II is a crucial enzyme involved in KYNA synthesis that uses KYN as a substrate. The crystal structure of the native complex of KAT II with KYN (Han et al. 2008) provided an important molecular basis for a comprehensive understanding of the substrate binding and catalysis in KAT II, thus enabling us to study the possible binding of ARBs (i.e., irbesartan, losartan, and telmisartan) to this enzyme. Docking simulations suggest that all studied ARBs bind to the KAT II active site. In addition, all ligands interact mostly with the same amino acids, including residues indicated for the KYN complex with KAT II (PDB ID: 2R2N). Finally, a higher number of hydrogen bonds are suggested for losartan, the compound experimentally determined to be the most potent inhibitor among tested ARBs.

Most studies on the pathogenesis of arterial hypertension have focused primarily on the peripheral mechanisms of blood pressure regulation, with lesser interest on the central nervous system. Among known pressor agents, AT-II and GLU play pivotal roles in the brain centers involved in blood pressure control in both normotensive and spontaneously hypertensive rats (SHR) (Muratani et al. 1991). Moreover, the location of AT_1_R in the central nervous system is strongly related to the cardiovascular regulation centers (Tagawa et al. 2000). The link between brain angiotensinergic and glutamatergic signaling was presented by Vieira et al. (2010). The major sympathetic output pathway for the tonic and reflex control of blood pressure, which uses GLU as the transmitter, arises from the rostral ventrolateral medulla (RVLM) (Colombari et al. 2001). Injection of AT-II into the RVLM of unanesthesized rats was shown to exaggerate pressor response to GLU. Administration of losartan into the RVLM reduced an increase in blood pressure caused by both GLU and AT-II (Vieira et al. 2010). Additionally, it is speculated that AT-II takes part in GLU pressor responses by presynaptic increase of GLU input into the RVLM (Kumagai et al. 2012).

Referring to this, KYNA (GLU antagonist) is claimed to be a hypotensive agent. Mills et al. (1990) reported that intrathecal KYNA administration decreased blood pressure, especially in anesthetized SHR and stroke-prone spontaneously hypertensive rats (SPR), with less noticeable effect in normotensive rats. What is more, lower KYNA content and decreased brain KAT activity in SHRs were observed (Kapoor et al. 1994). Ito et al. (2000) showed that KYNA injected into the RVLM of anesthetized SHR effectively reduced mean arterial pressure. The role of KYNA in blood pressure control was further emphasized by the discovery of a missense KAT I mutation E61G, which accounts for the reduced activity of KAT I as well as decrease in KYNA production in SHR (Kwok et al. 2002). Additionally, Mizutani et al. (2002) presented in SHR brainstem a higher expression of kynureninase, another enzyme involved in KYN degradation. Since the increased expression of kynureninase in SHR is thought to decrease the KYN level (Mizutani et al. 2002) and KYN is a precursor of KYNA, a decreased KYNA level can be expected in hypertensive rats. Interestingly, both an increase of mean arterial pressure and of splanchnic sympathetic nerve activity, evoked by AT-II administration into RVLM, were reduced by local administration of candesartan as well as KYNA (Kido et al. 2004). Considering the hypotensive activity of KYNA in the brain, the fact that all tested ARBs decreased the synthesis of this GLU antagonist is unexpected.

If ARBs decrease KYNA content in the brain and KYNA exerts neuroprotective and anticonvulsant activity (Schwarcz et al. 1987), an intensification of neurodegenerative processes and proconvulsant action of ARBs should be expected. To the contrary, ARBs are reported to be neuroprotective and anticonvulsant. Telmisartan, candesartan, losartan, and valsartan significantly reduced GLU-induced neuronal injury and apoptosis in cultured rat primary cerebellar granule cells (Wang et al. 2014). Losartan prevented neuronal loss and inhibited cognitive impairment in the pilocarpine-induced status epilepticus in rats (Sun et al. 2015) and exerted neuroprotection in the CA1 area of the hippocampus in the kainate model of temporal lobe epilepsy in rats (Tchekalarova et al. 2014). Moreover, losartan decreased seizure severity in Wistar audiogenic rats (Pereira et al. 2010) and prevented the development of delayed recurrent spontaneous seizures in two rat models of vascular injury (Bar-Klein et al. 2014).

In opposition to this, an elevated content of KYNA was linked with AD occurrence. Baran et al. (1999) reported significant KYNA increase in the putamen and caudate nucleus of AD brain, compared to other brain regions. In addition, this elevated KYNA level correlated with a significant increase in KAT I activity in both nuclei (Baran et al. 1999). Malkova et al. (2015) showed that intracerebral KYNA infusion impaired object recognition memory in macaques. Importantly, reduction of brain KYNA by PF-04859989, a brain-penetrable inhibitor of KAT II, improved cognitive function in rodents and nonhuman primates (Kozak et al. 2014).

In this study, ARBs inhibited KAT II activity and reduced the production of KYNA in rat cortical slices. According to the hypothesis that KYNA produces cognitive impairment, it can be expected that ARBs would positively affect the memory processes. Indeed, losartan improved cerebrovascular function in a mouse model of AD (Papadopoulos et al. 2017). Danielyan et al. (2010) have proved in a transgenic mouse model of AD that losartan given intranasally exerts a neuroprotective effect in concentrations much lower than that needed to decrease blood pressure. Moreover, enhancing memory effects were observed in humans treated with ARBs. Losartan improved cognitive function, mainly immediate and delayed memory in elderly hypertensive humans (Fogari et al. 2003) and in healthy young adults (Mechaeil et al. 2011). Accumulated data unequivocally indicate the beneficial effect of ARBs in memory impairment. However, the mechanism of such ARBs’ action is unknown. Our results imply that the decrease in KYNA production by ARBs may be responsible for the improving effect of these drugs on cognition.

Apart from memory improvement, ARBs may be beneficial in the treatment of psychotic disorders by decreasing KYNA production. High KYNA content, especially in the central nervous system, has been reported in patients with schizophrenia (Plitman et al. 2017). The reason for such an observation is unknown. One of the possible explanations is the involvement of RAS. It has been shown that RAS hyperactivity results in the alteration of central dopaminergic neurotransmission (Labandeira-García et al. 2014). The effect of ARBs was evaluated in drug induced animal schizophrenia models. Marchese et al. (2016) reported that losartan given intracerebroventricularly partially prevented the impairing effect of amphetamine in the inhibitory avoidance response of Wistar rats. In addition, losartan diminished amphetamine-induced hyperactivity in Wistar rats (Paz et al. 2014). Thus, it can be postulated that the antipsychotic effects of ARBs are linked with reduced brain KYNA concentration. To support this hypothesis, selective cyclooxygenase-2 (COX-2) inhibitors have also been proven to lower KYNA concentration in rat brain in vitro (Schwieler et al. 2006), as well as reduce amphetamine-induced behavioral changes in rats (El-Sayed El-Sisi et al. 2016). As a result, celecoxib is postulated as an adjunct therapy for patients with schizophrenia (Müller et al. 2010).

This study reports for the first time that ARBs inhibit KAT II activity and reduce KYNA production in cortical slices. The decrease of KYNA production in cortical slices can be explained by the inhibition of KAT II activity. Since the activity of KATs was investigated in partially purified enzymes, it can be concluded that the investigated ARBs, irbesartan, losartan, and telmisartan, are KAT inhibitors. This statement is further supported by our docking simulations which suggest that all studied ARBs bind to the KAT II active site.

Experimental data suggest that all analyzed ARBs can reach the central nervous system after peripheral administration (Zhuo et al. 1994; Culman et al. 1999; Kishi et al. 2012). Thus, it can be concluded that all examined ARBs can reach the central nervous system after systemic administration and affect KYNA production in the brain cortex.

This study has some limitations. Among the analyzed ARBs, only losartan potassium is water soluble, whereas irbesartan and telmisartan were dissolved in DMSO. Because of the limited solubility, the influence of telmisartan on KYNA production was examined up to 0.5 mM concentration.

In conclusion, the obtained results demonstrate that ARBs decrease KYNA synthesis in the brain cortex in vitro by inhibition of KAT II. In addition, we suggest that each studied ARB may bind to the KAT II active site, inhibit enzyme activity, and subsequently block KYNA production. Further in vivo studies are needed to confirm the presented in vitro findings.

## Electronic supplementary material

ESM 1 (DOCX 3350 kb)

## Abbreviations

ACE: Angiotensin converting enzyme
AD: Alzheimer’s disease
AMPA: α-Amino-3-hydroxy-5-methyl-4-isoxazolepropionic acid
ARBs: Angiotensin II type 1 receptor blockers
AT_1_R: Angiotensin II type 1 receptors
AT-II: Angiotensin II
COX-2: Cyclooxygenase-2
DMSO: Dimethyl sulfoxide
GLU: Glutamate
GPR35: G protein-coupled receptors 35
HPLC: High-performance liquid chromatography
KAT: Kynurenine aminotransferases
KYN: Kynurenine
KYNA: Kynurenic acid
NMDA: *N*-methyl-d-aspartate
PMP: 4′-Deoxy-4′-aminopyridoxal-5′-phosphate
RAS: Renin-angiotensin system
RVLM: Rostral ventrolateral medulla
SHR: Spontaneously hypertensive rats
SPR: Stroke-prone spontaneously hypertensive rats
