# Supplementary material for: Angiotensin II Type 1 Receptor Blockers Inhibit KAT II Activity in the Brain—Its Possible Clinical Applications
Source: Neurotox Res. 2017 Jul 21;32(4):639–48. doi: 10.1007/s12640-017-9781-2 (PMC5602025; doi:10.1007/s12640-017-9781-2)
Supplement: Supplementary file 1 — (DOCX 3350 kb) [file 12640_2017_9781_MOESM1_ESM.docx]

**Supplementary Materials**

**Angiotensin II type 1 receptor blockers inhibit KAT II activity in the brain – its possible clinical applications**

**Izabela Zakrocka^1^, Katarzyna M. Targowska-Duda^2^, Artur Wnorowski^2^,
Tomasz Kocki^1^, Krzysztof Jóźwiak^2^, Waldemar A. Turski^1^**

^1^Department of Experimental and Clinical Pharmacology, Medical University of Lublin, Lublin, Poland

^2^Department of Biopharmacy, Medical University of Lublin, Lublin, Poland

**Table of contents**

[1. Supplementary material and methods 2](#_Toc484074139)

[1.1 Microarray datamining 2](#_Toc484074140)

[2. Supplementary results 3](#_Toc484074141)

[2.1 Expression pattern on KAT-coding genes in brain cerebral cortex 3](#_Toc484074142)

[2.2 ARBs effect on the expression of KATs in rat 3](#_Toc484074143)

[Supplementary references 4](#_Toc484074144)

[Supplementary tables 5](#_Toc484074145)

[Supplementary figures 9](#_Toc484074146)

# Supplementary material and methods

## Microarray datamining

Data from previously published microarray experiments were extracted using Genevestigator software using *Anatomy* and *Perturbations* tools (Hruz et al. 2008). For anatomy-based search, only experiments carried out on cerebral cortex and on its substructures were included for the analysis. For the analysis of rat genes, only control animals were included. For the analysis of human gene expression, only data from healthy patients were extracted. Experiments regarding the effects of ARBs on the expression of KAT-coding genes were identified based on the search for "sartan" phrase in experiment description. Only experiments focused on circulatory and nervous systems with more than 2 data points per set were included for the analysis.

# Supplementary results

## Expression pattern on KAT-coding genes in brain cerebral cortex

Reanalysis of publicly available microarray datasets revealed that genes coding for KATs are expressed in both rat and human cerebral cortex across multiple experimental conditions (Fig. S2). *Aadat* gene coding for rat KAT II enzyme and its human orthologue were expressed at medium level across multiple experimental conditions. Genes coding for other isoforms of the enzyme were expressed at either similar or higher level compared to KAT II-coding gene.

## ARBs effect on the expression of KATs in rat

To test whether ARBs can downregulate the expression of KAT II and other KATs in rats the expression of *Kyat1*, *Aadat*, *Kyat3* and *Got2* genes was analyzed in previously published (Adler et al. 2016; Gusenleitner et al. 2014; Zhou et al. 2006) microarray datasets. These genes code for KAT I, KAT II, KAT III and KAT IV, respectively. We observed that candesartan, losartan, and valsartan caused no significant change in the expression of KAT-coding genes in rat heart (Fig. S3A). Chronic administration of candesartan did not affect the expression of KAT-coding genes in brain microvessels in a significant manner (Fig. S3B). There were no available data on the influence of ARBs on the KATs expression in the brain.

# Supplementary references

Adler M, Ramm S, Hafner M, Muhlich JL, Gottwald EM, Weber E, Jaklic A, Ajay AK, Svoboda D, Auerbach S, Kelly EJ, Himmelfarb J, Vaidya VS (2016) A quantitative approach to screen for nephrotoxic compounds in vitro. J Am Soc Nephrol 27:1015-28.

Gusenleitner D, Auerbach SS, Melia T, Gómez HF, Sherr DH, Monti S (2014) Genomic models of short-term exposure accurately predict long-term chemical carcinogenicity and identify putative mechanisms of action. PLoS One 9:e102579.

Hruz T, Laule O, Szabo G, Wessendorp F, Bleuler S, Oertle L, Widmayer P, Gruissem W, Zimmermann P (2008) Genevestigator v3: a reference expression database for the meta-analysis of transcriptomes. Adv Bioinformatics 2008:420747.

Zhou J, Pavel J, Macova M, Yu ZX, Imboden H, Ge L, Nishioku T, Dou J, Delgiacco E, Saavedra JM (2006) AT1 receptor blockade regulates the local angiotensin II system in cerebral microvessels from spontaneously hypertensive rats. Stroke 37:1271-6.

# Supplementary tables

**Table 1.** Residues involved in the binding of each ARB, KYN, and BFF-122 (KAT II inhibitor) to human KAT II active site. Residues interacting with KYN as well as common residues for KYN and other ligands are shown in green.

| Compound | Binding site | Hydrogen Bonds | Salt bridge | Hydrogen bonds with co-factor |
| --- | --- | --- | --- | --- |
| KYN  (PDB ID: 2R2N) | Ile19 (A)  Arg20 (A)  Gly39 (A)  Leu40 (A)  Tyr74 (A)  Leu293 (A)  Tyr142 (B)  Ser143 (B)  Asn202 (B)  Tyr233 (B)  Phe355 (B)  Phe387 (B)  Arg399 (B) | Asn202  Tyr74  Ser142  Ser143 | Arg399 | No |
| PMP (Co-factor)  (PDB ID: 2R2N) | Gly39 (A)  Tyr74 (A)  Leu293 (A)  Gly116 (B)  Ser117 (B)  Gln118 (B)  Leu121 (B)  Tyr195 (B)  Val197 (B)  Asp230 (B)  Pro232 (B)  Ser260 (B)  Ser262 (B)  Arg270 (B) | Ser117  Gln118  Ser262  Arg270 | No | Not determined |
| KYN | Ile19 (A)  Arg20 (A)  Gly38 (A)  Gly39 (A)  Leu40 (A)  Tyr74 (A)  Leu293 (A)  Tyr142 (B)  Ser143 (B)  Gly144 (B)  Asn202 (B)  Tyr233 (B)  Phe355 (B)  Phe387 (B)  Arg399 (B) | Gly39  Ser142  Tyr142 | Arg399 | No |
| Losartan  Orientation 1 | Ser17 (A)  Ile19 (A)  Arg20 (A)  Gly39 (A)  Leu40 (A)  Pro41 (A)  Tyr74 (A)  Ser77 (A)  Gln289 (A)  Leu293 (A)  His294 (A)  Tyr142 (B)  Ser143 (B)  Gly144 (B)  Asn202 (B)  Tyr233 (B)  Lys263 (B)  Met354 (B)  Phe355 (B)  Leu382 (B)  Phe387 (B)  Arg399 (B) | Leu293  Ser17  Ser77  Arg20  Ser143  Asn202  Arg399  H_2_O | No | No |
| Losartan  Orientation 2 | Ser17 (A)  Ile19 (A)  Arg20 (A)  Thr23 (A)  Gly38 (A)  Gly39 (A)  Leu40 (A)  Pro41 (A)  Tyr74 (A)  Ser77 (A)  Gln289 (A)  Leu293 (A)  His294 (A)  Tyr142 (B)  Ser143 (B)  Gly144 (B)  Asn202 (B)  Tyr233 (B)  Lys263 (B)  Met354 (B)  Phe355 (B)  Leu382 (B)  Arg399 (B) | Ser17  Ser143  Tyr142  Asn2020  Tyr233  Gly39  Pro41  H_2_O | No | Two hydrogen bonds |
| Irbesartan  Orientation 1 | Ile19 (A)  Arg20 (A)  Gly39 (A)  Leu40 (A)  Pro41 (A)  Tyr74 (A)  Ser77 (A)  Gln289 (A)  Val290 (A)  Leu293 (A)  His294 (A)  Tyr142 (B)  Ser143 (B)  Gly144 (B)  Asn202 (B)  Tyr233 (B)  Lys263 (B)  Met354 (B)  Phe355 (B)  Phe387 (B)  Arg399 (B) | Ser17  Ser77  Arg20  Arg399  Asn202  H_2_O | No | No |
| Irbesartan  Orientation 2 | Pro16 (A)  Ser17 (A)  Ile19 (A)  Arg20 (A)  Thr23 (A)  Gly38 (A)  Gly39 (A)  Leu40 (A)  Pro41 (A)  Tyr74 (A)  Ser77 (A)  Gln289 (A)  Leu293 (A)  His294 (A)  Tyr142 (B)  Ser143 (B)  Gly144 (B)  Asn202 (B)  Tyr233 (B)  Lys263 (B)  Met354 (B)  Phe355 (B)  Leu382 (B)  Phe387 (B)  Arg399 (B) | Arg20  Arg399  Asn202  H_2_O | No | Two hydrogen bonds |
| Telmisartan | Pro16 (A)  Ser17 (A)  Ile19 (A)  Arg20 (A)  Gly39 (A)  Leu40 (A)  Tyr74 (A)  Ser75 (A)  Ser77 (A)  Gln289 (A)  Val290 (A)  Leu293 (A)  His294 (A)  Ala141 (A)  Tyr142 (B)  Ser143 (B)  Gly144 (B)  Asn202 (B)  Pro203 (B)  Tyr233 (B)  Met354 (B)  Phe355 (B)  Phe387 (B)  Arg399 (B) | Pro16  Ser17  Ser143  Arg399 | No | No |

# Supplementary figures


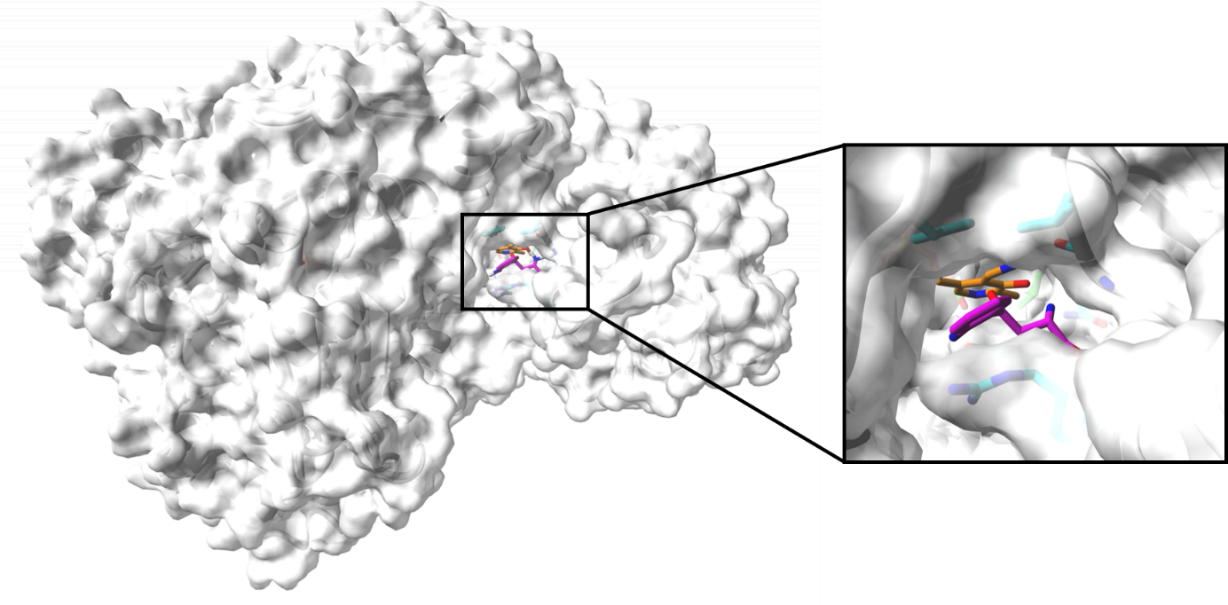


**A**


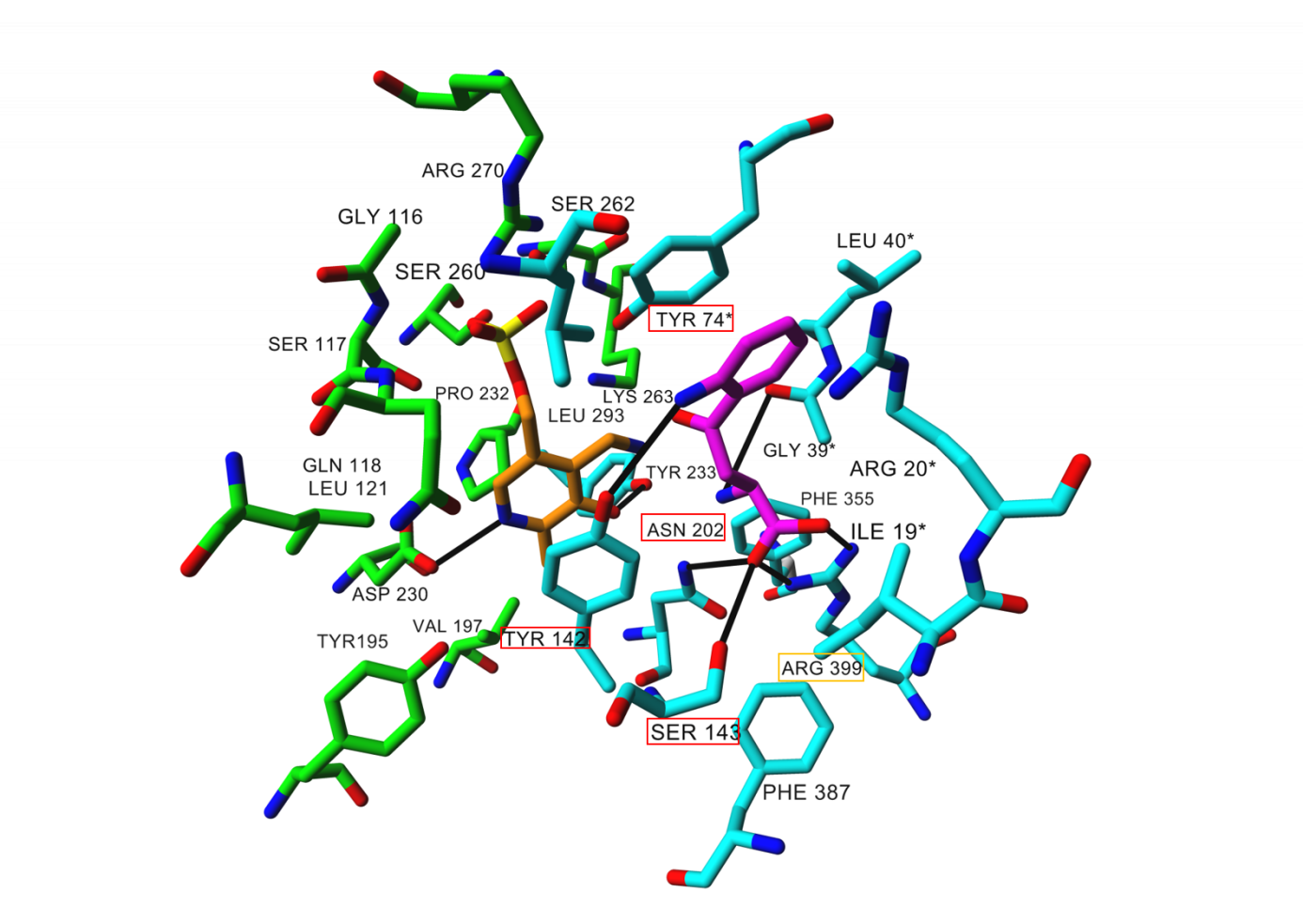


**B**

**Fig. S1.** Binding site for KYN and co-factor PMP at the crystal structure of KAT II (PDB ID: 2R2N) presented as a molecular surface (panel A). Detailed view of residues involved in KYN and PMP binding at KAT II (panel B). Ligand and co-factor are rendered in stick mode, residues involved in KYN (magenta) and PMP (orange) binding are shown in cyan and green, respectively (panel A and B). Black solid lines indicate hydrogen bonds as well as salt bridges formed between KYN and amino acid residues. In addition, all residues involved in hydrogen bonding and salt bridge are marked in red and yellow, respectively. Oxygen atoms are colored red, nitrogen blue, phosphorus yellow, and chlorine green. All hydrogen atoms are hidden.


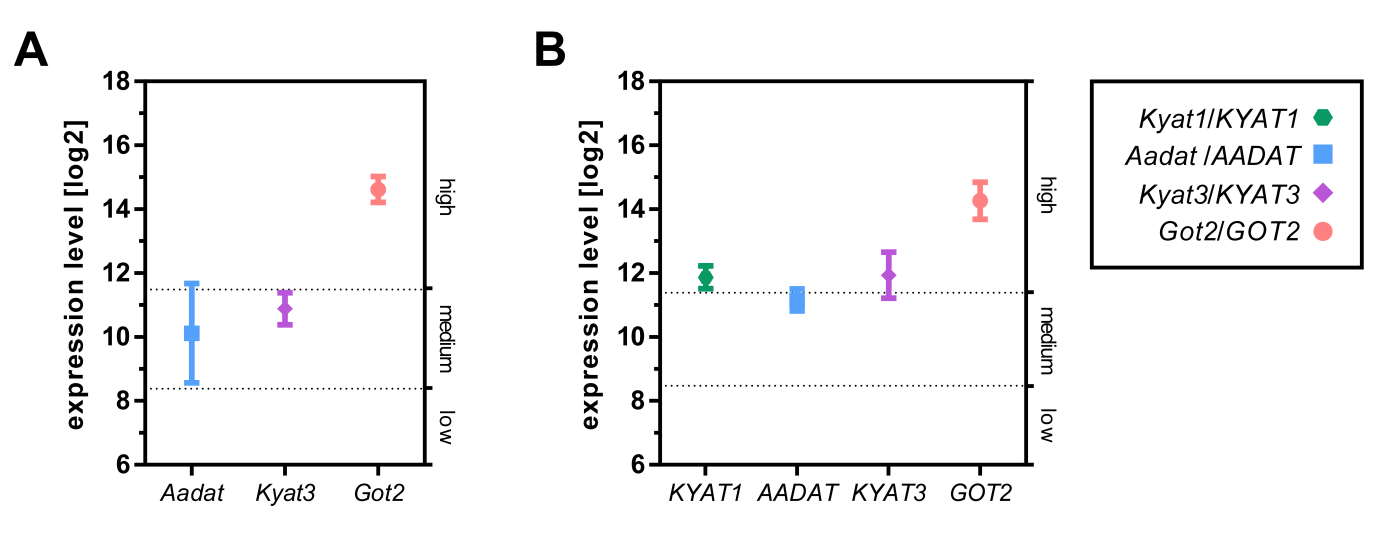


**Fig. S2. Expression levels of KAT-coding genes in cerebral cortex (A)** Data on rat gene expression were extracted from previously published results of microarray experiments carried out on Affymetrix Rat Genome 230 2.0 Array (n = 127). *Aadat*, *Kyat3* and *Got2* genes code for KAT II, KAT III and KAT IV, respectively. Probe for *Kyat1* gene coding for rat KAT I enzyme was not represented on the selected microarray platform. Data originate from GEO database (GSE2870, GSE2880, GSE4776, GSE4753, GSE7323, GSE9798, GSE9798, GSE13428, GSE13624, GSE14269, GSE14505) and ArrayExpress database (E-MEXP-553). **(B)** Expression levels of human genes were from Affymetrix Human Genome U133 Plus 2.0 Array (n = 617). Data were extracted from GEO (GSE3526, GSE4757, GSE7307, GSE21138, GSE21935, GSE17612, GSE13564, GSE11882, GSE16759, GSE35864, GSE33010, GSE50161, GSE53890, GSE30643, GSE53987, GSE43346) and ArrayExpress (E-MEXP-2280, E-TABM-20) databases. Dotted horizontal lines indicate the expression value of a given probe in relation to all genes over all samples available through Genevestigator software for the platform in use. Probes located below the lower dotted line fall within the first quartile, and thus are considered "low". Probes with values between the two dotted lines correspond to the interquartile range and are characterized by "medium" expression level. Probes within the upper part of the graph correspond to the top 25% of probes, thus are considered "high" by the means of expression level.


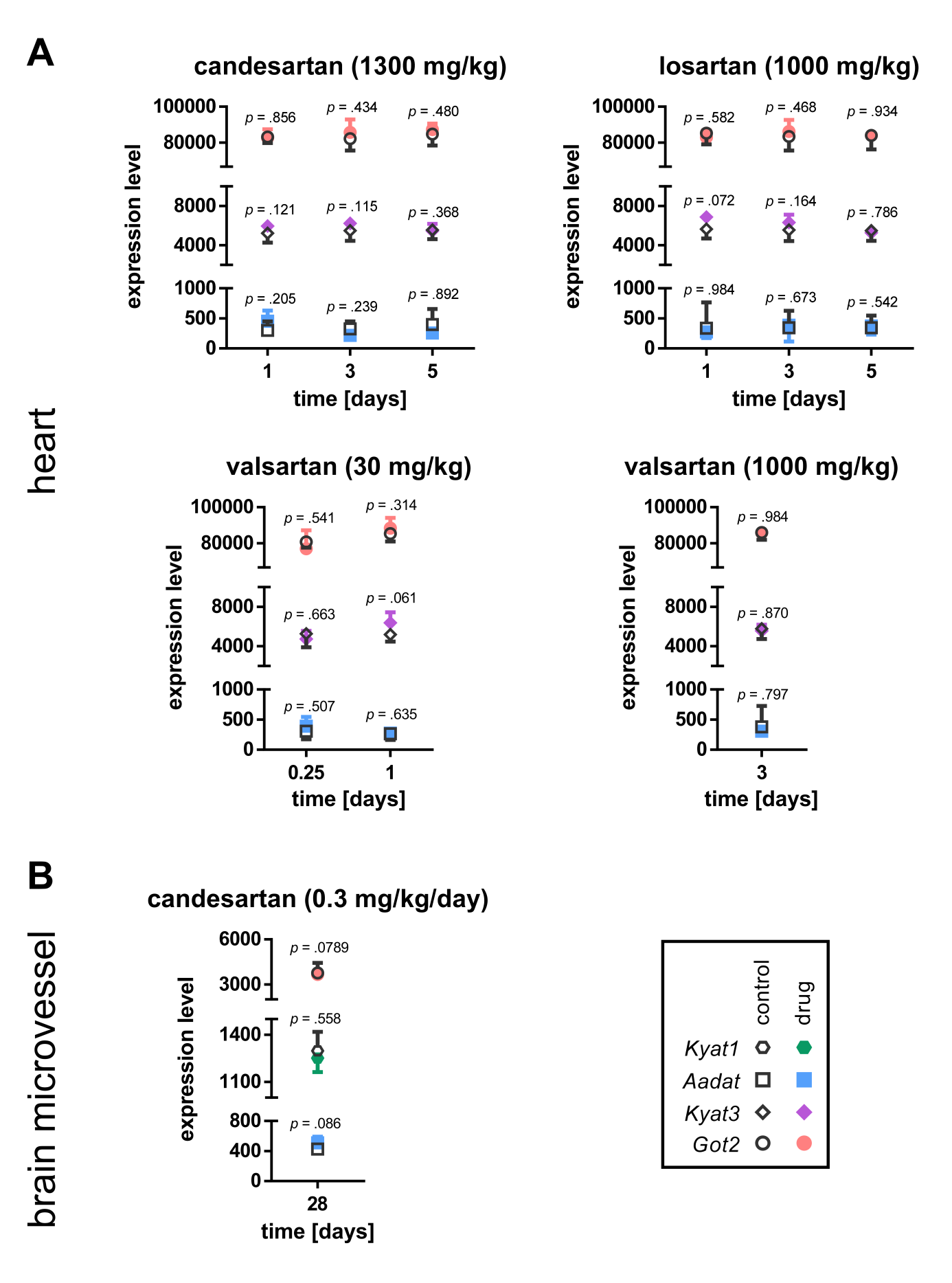


**Fig. S3. Effect of ARBs on the expression of KATs at different body sites in rats** **(A)** Changes in *Aadat*, *Kyat3*, and *Got2* expression in heart in response to candesartan (1300 mg/kg), losartan (1000 mg/kg), and valsartan (30 or 1000 mg/kg). **(B)** Changes in *Kyat1*, *Aadat*, and *Got2* in microvessels of rat brain in response to candesartan (0.3 mg/kg/day). The microarray data were obtained from previously published works deposited in GEO database (accession numbers: GSE57822, GSE57800, GSE57816, GSE57811, GSE2739). Data extraction was carried out using Genevestigator software. Data originate from GeneChip Rat Genome 230 and GeneChip Rat Genome U34 microarray platform**.**
